# Endothelial cell ferroptosis mediates monocrotaline-induced pulmonary hypertension in rats by modulating NLRP3 inflammasome activation

**DOI:** 10.1038/s41598-022-06848-7

**Published:** 2022-02-23

**Authors:** Shan-Shan Xie, Yan Deng, Sheng-lan Guo, Jia-quan Li, Ying-chuan Zhou, Juan Liao, Dan-dan Wu, Wei-Fang Lan

**Affiliations:** 1 Department of Ultrasound, First Affiliated Hospital of Guangxi Medical University, 6 Shuang yong Road, Nanning, 530021 People’s Republic of China; 2 Experimental Centre of Guangxi Medical University, Nanning, People’s Republic of China; 3 Department of Pathology, First Affiliated Hospital of Guangxi Medical University, Nanning, People’s Republic of China; 4 Department of Echocardiography of Cardiovascular Disease Institute, First Affiliated Hospital of Guangxi Medical University, 6 Shuang yong Road, Nanning, 530021 People’s Republic of China

**Keywords:** Cardiovascular diseases, Cardiovascular biology

## Abstract

Inflammation triggers pulmonary vascular remodelling. Ferroptosis, a nonapoptotic form of cell death that is triggered by iron-dependent lipid peroxidation and contributes to the pathogenesis of several inflammation-related diseases, but its role in pulmonary hypertension (PH) has not been studied. We examined endothelial cell ferroptosis in PH and the potential mechanisms. Pulmonary artery endothelial cells (PAECs) and lung tissues from monocrotaline (MCT)-induced PH rats were analysed for ferroptosis markers, including lipid peroxidation, the labile iron pool (LIP) and the protein expression of glutathione peroxidase 4 (GPX4), ferritin heavy chain 1 (FTH1) and NADPH oxidase-4 (NOX4). The effects of the ferroptosis inhibitor ferrostatin-1 (Fer-1) on endothelial cell ferroptosis and pulmonary vascular remodelling in MCT-induced rats were studied in vitro and in vivo. Ferroptosis was observed in PAECs from MCT-induced PH rats in vitro and in vivo and was characterized by a decline in cell viability accompanied by increases in the LIP and lipid peroxidation, the downregulation of GPX4 and FTH1 expression and the upregulation of NOX4 expression. High-mobility group box 1 (HMGB1)/Toll-like receptor 4 (TLR4)/NOD-like receptor family pyrin domain containing 3 (NLRP3) inflammasome signalling was measured by western blotting. These changes were significantly blocked by Fer-1 administration in vitro and in vivo. These results suggest that Fer-1 plays a role in inhibiting ferroptosis-mediated PAEC loss during the progression of PH. The ferroptosis-induced inflammatory response depended on the activation of HMGB1/TLR4 signalling, which activated the NLRP3 inflammasome in vivo. We are the first to suggest that pulmonary artery endothelial ferroptosis triggers inflammatory responses via the HMGB1/TLR4/NLRP3 inflammasome signalling pathway in MCT-induced rats. Treating PH with a ferroptosis inhibitor and exploring new treatments based on ferroptosis regulation might be promising therapeutic strategies for PH.

## Introduction

Pulmonary hypertension (PH) is a fatal and complex vascular disease that leads to right-side heart failure and, eventually, death^1, 2^. Although numerous therapeutic options for PH have been explored in the past two decades, currently available therapies remain essentially palliative^3, 4^. Further studies are needed to understand the underlying key regulators of this type of pulmonary vascular remodelling, which may help in designing new and effective approaches for PH treatment^3^. Strong evidence suggests that inflammation and immunity play critical roles in the pathogenesis of PH. However, the precise molecular mechanisms that orchestrate inflammation in the context of PH pathogenesis remain unclear.

Ferroptosis is a novel form of nonapoptotic cell death that is dependent on iron and reactive oxygen species (ROS). The characteristics of ferroptosis are cell volume shrinkage and mitochondrial membrane thickening^5, 6^. Ferroptosis results in the release of inflammatory cytokines and danger-associated molecular patterns (DAMPs), which are involved in many inflammation-related disease processes^7^, such as tumours^8^, kidney diseases^9, 10^, and heart transplantation^11^. The progression of PH can be attributed to increased ROS production^12^. ROS are important in endothelial dysfunction and damage and are critical initial players in vascular inflammation^13, 14^. Endothelial cell ferroptosis has been observed in atherosclerotic tissues and is involved in the progression of atherosclerosis^15^. Damage to pulmonary artery endothelial cells (PAECs) was crucial in the early onset of PH^16^. Thus, we hypothesize that the promotion of ROS generation leads to endothelial ferroptosis, which initiates inflammatory reactions and is involved in the progression of PH.

High-mobility group box 1 (HMGB1) is a well-known, abundant and typical DAMP. Previous studies have confirmed that HMGB1 is released from ferroptotic cells^8, 17, 18^. The biological effects of HMGB1 depend on its subcellular localization and posttranslational modifications. When cell damage or death occurs, HMGB1 is released into the extracellular environment and is involved in a range of processes, such as inflammation, proliferation, and migration^19^. HMGB1 exerts its biological effects by binding to Toll-like receptor 4 (TLR4), a critical element of the innate immune system, subsequently triggering a chain of downstream signalling cascades^20, 21^. Previous studies have shown that targeting HMGB1 or TLR4 in PH animal models protects against PH^22, 23^.

The NOD-like receptor family pyrin domain containing 3 (NLRP3) inflammasome is an extensively studied inflammasome^24, 25^. Recent studies have shown that the NLRP3 inflammasome is activated in macrophages in the lung in animal models of PH and that inhibiting the inflammasome ameliorates monocrotaline (MCT) or hypoxia-induced PH^26, 27^. The NLRP3 inflammasome needs to be primed by activating TLR4. The NLRP3 inflammasome is involved in the production of proinflammatory cytokines, including interleukin-1β (IL-1β) and IL-18, and plays a deleterious role in PH^25^. In this study, we hypothesized that PAEC ferroptosis caused HMGB1 release and subsequent interaction with TLR4 in macrophages and then activated the NLRP3 inflammasome to initiate vascular inflammation.

MCT-induced PH in rats is one of the most classical and widely used in vivo models of PH that is to study severe PH characterized by acute or subacute pulmonary vasculature injury^28, 29^ and is an essential model for understanding PH pathophysiology and for the discovery and development of novel therapies^28, 29^. Although this model cannot completely recapitulate the biological properties of human PH, the activation of oxidative stress^30, 31^, PAEC dysfunction^32^ and inflammatory response activation can still provide some insights into the underlying pathogenesis of PH^30, 31^. Here, we investigated whether ferroptosis was present in PAECs and elucidated the contribution of PAEC ferroptosis to vascular pathology in MCT-induced PH.

## Materials and methods

### Materials

Ferrostatin-1 (Fer-1, a selective inhibitor of ferroptosis), HMGB1 and TAK-242 (a TLR4 inhibitor) were purchased from Selleckchem (Shanghai, China). Dihydrochloride (DAPI), a Cell Counting Kit-8 (CCK-8) assay kit, collagenase and MCT were obtained from Sigma–Aldrich (St. Louis, MO, USA). Primary antibodies against α-smooth muscle actin (α-SMA) and GAPDH were obtained from Boster Biological Technology (Wuhan, China). Primary antibodies against TLR4, nicotinamide adenine dinucleotide phosphate (NADPH) oxidase 4 (NOX4), glutathione peroxidase 4 (GPX4), and ferritin heavy chain 1 (FTH1) were obtained from Cell Signaling Technology (Beverly, MA, USA). Primary antibodies against NLRP3, ASC, pro-caspase1 and caspase1 were also obtained from Cell Signaling Technology (Beverly, MA, USA). The iron assay kit and HMGB1, IL-1β, IL-18, and tumour necrosis factor alpha (TNF-α) enzyme-linked immunosorbent assay (ELISA) kits were obtained from R&D Systems (Minneapolis, MN, USA), and the primary antibody against CD31 was obtained from Novus Biologicals Inc. (Colorado, USA).

### Animals and groupings

All studies were conducted according to the Guidelines for the Care and Use of Experimental Animals (NIH Publication No. 85–23, revised 1996), approved by the Guangxi Medical University Animal Ethics Committee, and carried out in compliance with the ARRIVE guidelines (http://www.nc3rs.org.uk/page.asp?id=1357). Pathogen-free inbred male Sprague–Dawley rats (220–250 g) were obtained from the Experimental Laboratory Animal Centre of Guangxi Medical University (Nanning, China). The rats were placed in housing conditions with standard room temperature (21–22 °C) and humidity (60–65%). Food and water were available ad libitum. The PH rat model was established by a single subcutaneous injection of MCT (60 mg/kg). MCT was dissolved in 1 N HCl, neutralized to pH 7.4 with the addition of 0.5 N NaOH and then diluted with distilled water. To examine the effect of ferroptosis inhibition in on progression of PH, Fer-1, a widely used selective inhibitor of ferroptosis, was administered to the MCT-induced rats^32–35^. A total of 48 male rats were randomly assigned to four experimental groups (n = 12/group): (1) saline + vehicle-treated control group (sham group), (2) saline + Fer-1-treated (a specific inhibitor of ferroptosis, 2 mg/kg/d) group (sham + Fer-1 group), (3) MCT + vehicle (normal saline)-treated group (MCT group), and (4) MCT + Fer-1-treated (2 mg/kg/d) group (MCT + Fer-1 group). The dose of Fer-1 used in the study was based on a previous report^36^. The first doses of Fer-1 and vehicle were administered by oral gavage 1 h before MCT administration. Then, the treatments were administered once per day for 4 weeks after MCT injection. Body weight was measured once per week to adjust the dose accordingly.

### Echocardiography

A previous study revealed that changes in the pulmonary vasculature, haemodynamic alterations and right ventricular (RV) remodelling were evident in rats at 28 days after MCT administration^23^. Thus, echocardiography and haemodynamic evaluations were performed at this time. The rats were anaesthetized with ketamine (75 mg/kg). Echocardiography was performed using an HP 7500 system with a 12S transducer (Philips, Hewlett-Packard Co., Andover, MA, USA). Tricuspid annular plane systolic excursion (TAPSE), RV end diastolic dimension (RVEDD) and right ventricular ejection fraction (RVEF) were determined via echocardiography as previously described^27, 37^. Measurements were recorded from 10 consecutive beats and were used to normalize beat-to-beat variations.

### Haemodynamic measurements and tissue processing

Using a closed chest approach, RV systolic pressure (RVSP) was measured by direct RV puncture. A 21G puncture needle was connected to a pressure sensor (ALCB10 Heart Function Analysis System; Shanghai Alcott Biotech CO. LTD, China) and used to measure RVSP. When the RV outflow tract and pulmonary valve stenosis were excluded by echocardiography, the pulmonary artery systolic pressure (PASP) was equivalent to the RVSP^23, 38^. After the haemodynamic measurements were collected, the animals were euthanized; the lungs and hearts were rapidly excised and weighed. The dry weights of the right ventricular free wall (RV) and the left ventricle (LV) plus septum (LV + S) were measured, and then the RV/LV + S was calculated. Part of the RV and inflated left lung tissues from all experimental groups were rinsed in chilled saline, perfused with 4% paraformaldehyde and embedded in paraffin for 24 h before histopathological examinations were performed. The left lung and RV tissues were immediately stored in liquid nitrogen at − 70 °C. The tissues were used for ELISA, fluorescence staining with immunoglobulin, and western blotting.

### Histological examination

Haematoxylin–eosin staining was used to stain lung Sects. (5 μm), and the medial wall thickness was calculated. Occlusion was calculated in intralacinar pulmonary vessels with outer diameters of 50–100 μm by the following formula: (outer vessel area—luminal area)/(outer vessel area). For each animal, more than 10 randomly chosen vessels that were generally round or oval in shape were measured and averaged by an observer who was blinded to the experimental groups. All slides were observed and imaged using an Olympus BX51 microscope equipped with analysis software (Olympus, Tokyo, Japan). Images were analysed using Image-Pro Plus 6.0 (Media Cybernetics, Silver Spring, MD, USA). Two pathology experts who were blinded to the treatment groups randomly selected five fields from each section for analysis^39^.

### Immunohistochemistry and immunofluorescence analysis

To assess muscularization of the pulmonary vasculature, lung sections were paraffin-embedded and processed for immunohistochemistry using rabbit anti-α-SMA (1:150). Then, 3% H_2_O_2_ was added and incubated at room temperature for 5–10 min to eliminate endogenous peroxidase activity. The sections were rinsed with distilled water and soaked in phosphate-buffered saline (PBS) for 10 min. Normal goat serum (5–10%) was added and incubated at room temperature for 10 min. The working solution was added and incubated at 37 °C for 1–2 h or at 4 °C overnight. Appropriate amounts of biotin-labelled secondary antibody working solution were added and incubated at 37 °C for 10–30 min. Appropriate amounts of horseradish enzyme- or alkaline phosphatase-labelled Streptomyces ovalbumin working solution were added and incubated at 37 °C for 10–30 min. The colour was developed for 3–15 min (DAB or NBT/BCIP), and then the sections were fully rinsed in tap water, dyed again, dehydrated, made transparent, and sealed with carbon. The capillary density of the right ventricle is expressed as the number of CD31-positive spots (1:400, Novus Biologicals, US) per cross-sectional area. Nuclei were stained with DAPI. Images were captured using an Olympus DP80 microscope equipped with analysis software (Olympus, Tokyo, Japan). Images were examined using Image-Pro Plus 6.0 software (Media Cybernetics, USA). Fifteen slices from each animal were evaluated by two pathology experts who were blinded to the treatment groups.

### PAEC isolation

Rat PAECs from the sham group (12 rats) and MCT group (8 rats exposed to MCT for 4 weeks) were isolated for further study as previously described^40^. Briefly, rat lung parenchyma samples were rinsed with DMEM, cut into 1–2 mm^3^ pieces and digested for 60 min in collagenase (1 mg/ml) at 37 °C in a shaking incubator. The cellular digest was filtered through sterile mesh and centrifuged (400 × g for 7 min). After 5–7 days, cells exhibiting cobbled morphology were selected, treated with trypsin, and replated on gelatine-coated dishes. Next, the cells were labelled with fluorescein and acetylated low-density lipoprotein (AcLDL; Alexa Fluor 488-AcLDL; Invitrogen), and AcLDL-positive cells were sorted and collected by flow cytometry (FACS Aria; BD Biosciences, San Jose, CA). Cells were grown in a humidified atmosphere of 5% CO_2_ at 37 °C in DMEM supplemented with foetal bovine serum (FBS), 100 μg/ml streptomycin, 100 U/ml penicillin, 0.25 μg/ml amphotericin B, and 0.1 mM MEM with nonessential amino acids. PAECs were used in further experiments when they were nearly 70–80% confluent.

### Cell treatments

First, to examine the interactions between PAECs and macrophages, in vitro coculture models were used to study various PH-related events in Transwell chambers^41^. Macrophages (NR8383 cell line, Cell Bank of the Chinese Academy of Sciences, Shanghai, China) were seeded in the bottom well, while MCT-induced PAECs derived from PH rats were seeded on top of the insert. Cell-to-cell exchanges rely on soluble secretions. The PAECs were separated into the following three groups: normal control (NC) group cells were from sham group rats; model group cells were from MCT-induced PH rats; and Fer-1 group cells were from MCT-induced PH rats treated with Fer-1 (10 nM). Markers of ferroptosis were assessed in PAECs, TLR4 protein expression was evaluated in macrophages, and HMGB1 levels were measured in the cell culture medium. Second, to study HMGB1-induced NLRP3 inflammasome activation via TLR4 activation, the NR8383 cell line was divided into three groups: the negative group; the HMGB1 group, in which the NR8383 cell line was treated with HMGB1 (800 ng/mL); and the TAK-242 group, in which the NR8383 cell line was treated with HMGB1 (800 ng/mL) and a TLR4 inhibitor (TAK-242, 1 μM). TLR4 and NLRP3 inflammasome markers were assessed by western blotting. TNF-α, IL-18 and IL-1β levels were measured in the cell culture medium. The concentrations of Fer-1, HMGB1 and TAK-242 used were determined according to the manufacturer’s instructions.

### Cell counting kit-8 assay

CCK-8 (Dojindo Laboratory, Kumamoto, Japan) was used to determine PAEC viability according to the manufacturer's instructions. Briefly, cells were plated at a density of 2.0 × 10^5^ cells/well in 96-well plates. After the treatments, cell proliferation was assessed with a CCK-8 kit according to the manufacturer’s instructions. The relative amounts of cells were determined in triplicate based on the control cells.

### Iron assay

After the treatments, the cells were washed 3 times with PBS to remove free iron, and calcein-AM working solution was added and incubated at 37 °C for 30 min. After the cells were washed with PBS 3 times, a fluorescence microscope equipped with analysis software (Olympus, Tokyo, Japan) was used to observe the cells (λex = 490 nm, λem = 515 nm). Calcein-AM staining was used to visualize the labile iron pool (LIP) according to the manufacturer’s protocol (Catalogue number C326, Dojindo, Japan). The relative iron concentration in lung sample lysate was assessed with an iron assay kit (ab83366; Abcam) according to the manufacturer’s instructions.

### Lipid peroxidation assay

Lipid peroxidation was measured using a malondialdehyde (MDA) assay kit (Beyotime Biotech, Nanjing, China). In brief, cells were harvested by trypsinization, and cellular extracts were prepared by sonication in ice-cold RIPA buffer (Beyotime Biotech, Nanjing, China). After sonication, the lysed cells were centrifuged at 10,000 × g for 20 min to remove debris. The MDA levels and protein concentrations of the supernatants were determined. We used a protein assay kit (Bio–Rad Laboratories, Hercules, USA) to quantify the protein concentration. MDA levels were then normalized to milligrams of protein. The MDA level is expressed as the ratio of the absorbance value to that of the control cells^42^.

### Mitochondrial membrane potential assay

Mitochondrial membrane potential was examined using JC-1 according to the manufacturer’s instructions (Beyotime Biotech, Nanjing, China). Briefly, cells were cultured in 6-well plates at a density of 3 × 10^5^ cells per well. After 24 h, the cells were stained with JC-1 and observed by an Olympus BX51 microscope equipped with analysis software (Olympus, Tokyo, Japan). The excitation wavelength of JC-1 is 488 nm.

### Transmission electron microscopy (TEM)

PAECs were washed with PBS, fixed with 4% paraformaldehyde and buffered 2.5% glutaraldehyde for 2 h, and then washed with 0.1 M phosphate buffer containing 4% sucrose. Post fixation was performed with a 1% OsO_4_ solution in 0.1 M phosphate buffer for 90 min, dehydration was performed, and propylene oxide was added. The samples were infiltrated with epoxy resins, and the resin was polymerized at 60 °C for 72 h. Ultrathin sectioning was performed at a thickness of 70–80 nm, and the sections were stained with a double-contrast method using 2% uranyl acetate and Reynolds lead citrate solution. The sections were observed under a transmission electron microscope (H-7700; Hitachi High-Technologies, Tokyo, Japan).

### ELISA

Cell culture supernatants and rat lung tissue homogenates were analysed after centrifugation. The levels of HMGB1, TNF-α, IL-1β and IL-18 were measured using commercially available ELISA kits (R&D Systems, Minneapolis, MN, USA) according to the manufacturer's instructions.

### Western blot analysis

Snap-frozen whole lung samples were homogenized in phosphate buffer (150 mM NaCl, 50 mM Tris–HCl, 1% Triton X-100, pH 7.4, 1 mM EDTA, 0.1% SDS, and 1% sodium deoxycholate) with protease inhibitors and centrifuged at 12,000 rpm at 4 °C for 10 min to obtain cytosolic protein fractions. Equal amounts of protein were separated by 10% SDS–polyacrylamide gel electrophoresis and transferred onto nitrocellulose membranes. The membranes were incubated at 4 °C overnight with the following primary antibodies: GPX4 (1:200, sc-58607), FTH1, NOX4, TLR4, apoptosis-associated speck-like protein containing a caspase (ASC), pro-caspase1, caspase1, and NLRP3 (1:1000; all Cell Signaling Technology, USA). All antibodies were incubated overnight at 4 °C in 5% bovine serum albumin (BSA) in Tris-buffered saline with Tween (TBST). The membranes were washed and subsequently incubated with horseradish peroxidase-conjugated secondary antibodies (1:5000; ZSGB-Bio, Beijing, China) for 1 h at room temperature. The membranes were developed with enhanced chemiluminescence reagents, protein bands were visualized by exposure to X-ray film using a gel imaging system (UVP, Upland, CA), and the protein bands were quantified using ImageJ software (National Institutes of Health, Bethesda, MD). Band intensity was normalized to GAPDH levels.

### Statistical analysis

The data are expressed as the mean ± standard deviation (SD). All data were analysed using SPSS 17.0 software (SPSS Inc., Chicago, IL, USA). Comparisons of parameters between the two model groups before treatment were performed with unpaired Student’s t tests (which followed a normal distribution). Comparisons of parameters among more than two groups were performed by one-way analysis of variance (ANOVA), followed by the Newman–Keuls multiple comparison test for normally distributed data. Significant differences were defined as *P* < 0.05 (two-tailed).

## Results

### Ferroptosis is upregulated in PAECs from MCT-induced rats in vitro

To determine whether ferroptosis occurs in PAECs in MCT-induced PH, CCK-8 assays, lipid peroxidation measurement, cellular iron accumulation assays, and analysis of mitochondrial morphology and ferroptosis-related molecules were performed^6, 43–45^. To further verify ferroptosis in PAECs, Fer-1, a selective inhibitor, was used. Fer-1 has been shown to suppress ferroptosis in different cells and in preclinical models^32–35, 46^. The mechanism by which Fer-1 prevents cell death has been ascribed to the direct inhibition of lipid peroxidation by binding to the 15LOX/PEBP1 complex and suppressing the generation of peroxidised ETE-PE^47^.

**Figure 1:**
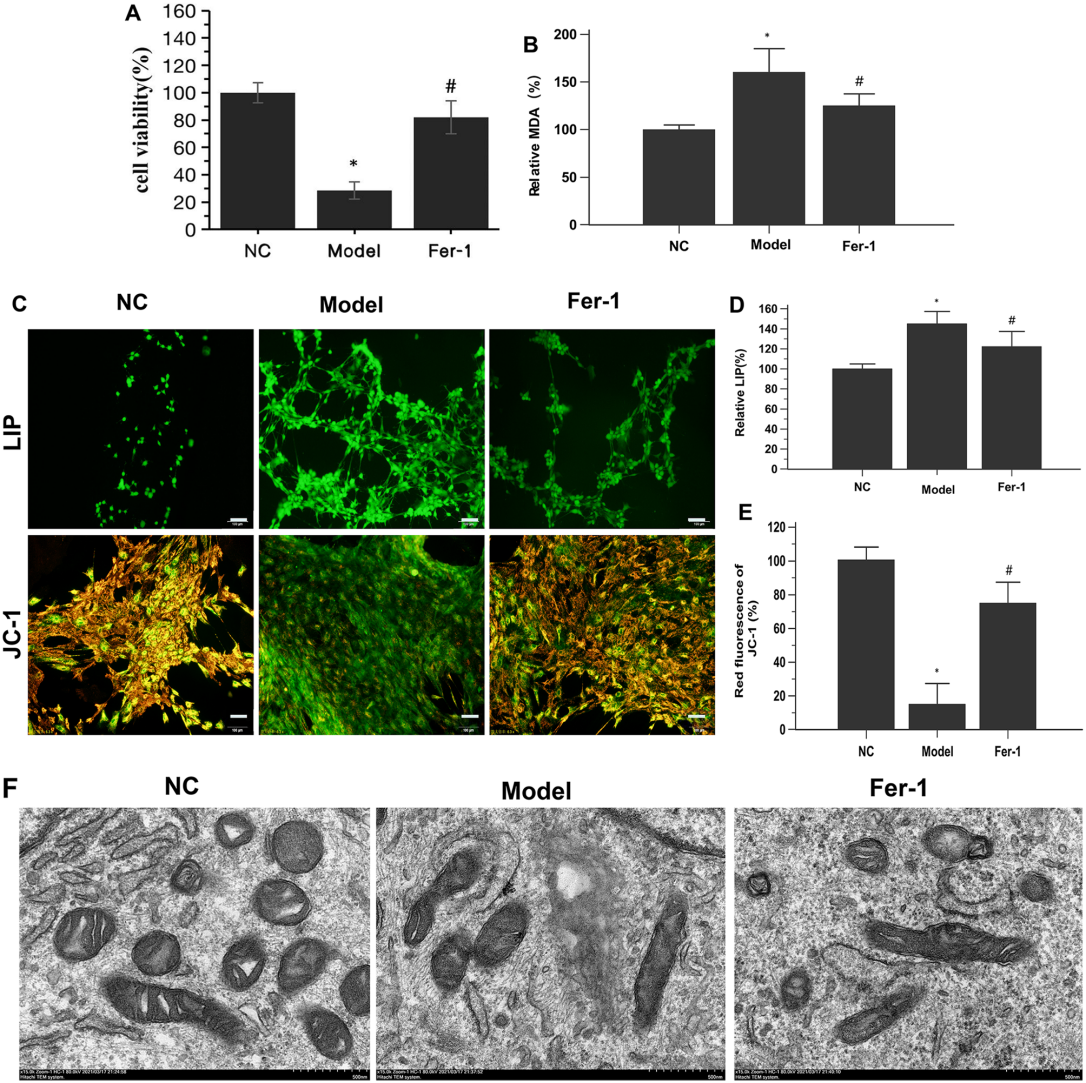
Ferroptosis occurs in PAECs derived from MCT-induced PH rats. (**A**) Fer-1 restored the viability of MCT-induced PH rat-derived PAECs. (**B**) Fer-1 reduced lipid peroxidation in MCT-induced PH rat-derived PAECs. (**C**) Representative LIP and JC-1-stained PAECs. Morphology was examined using light microscopy. Scale bar = 100 μm. (**D**) Quantification of the relative LIP value in the different groups of PAECs. (**E**) Quantification of the relative value of JC-1 red fluorescence in the different groups of PAECs. (**F**) Representative TEM images of PAECs in the different groups show mitochondrial morphology. PAECs in the model group showed mitochondrial damage with mitochondrial outer membrane rupture and mitochondrial crista disappearance. Fer-1 restored these changes. Scale bar = 500 nm. NC, normal control group; Model, model group; Fer-1, MCT + Fer-1 (10 nM, treated for 4 h) group. Each experiment was repeated 3 times, n = 3. The data represent the means ± SD. *P < 0.05 versus the NC group, #*P* < 0.05 versus the model group.

The CCK-8 assay results showed that the viability of PAECs in the model group was significantly decreased compared to that in the NC group (Fig. 1A). PAECs in the model group exhibited significant increases in MDA levels and the LIP, which are common markers of ferroptosis^45^, compared with those in the NC group. Importantly; these alterations were significantly reversed by the ferroptosis inhibitor Fer-1 (Fig. 1B–D).

Next, we assessed mitochondrial morphology with JC-1 and TEM. We used the JC-1 probe to detect mitochondrial membrane potential damage in PAECs, and we found that red fluorescence was reduced in PAECs from MCT-induced PH rats, while green fluorescence was increased (Fig. 1C and E), indicating mitochondrial damage. Mitochondria are major hubs of iron accumulation; specifically, iron overload typically causes mitochondrial dysfunction, as mitochondria are the targets of iron-mediated damage^44^. The TEM images showed that the PAECs in the NC group were intact and that the mitochondria were linear or granular with an integral bilayer membrane structure. In contrast, the PAECs in the model group were marked by decreased mitochondrial cristae, swollen mitochondria, and ruptured mitochondrial membranes (Fig. 1F), which are morphological features associated with ferroptosis^44, 48^. This mitochondrial feature was prevented in the model group of cells treated with Fer-1.

**Figure 2:**
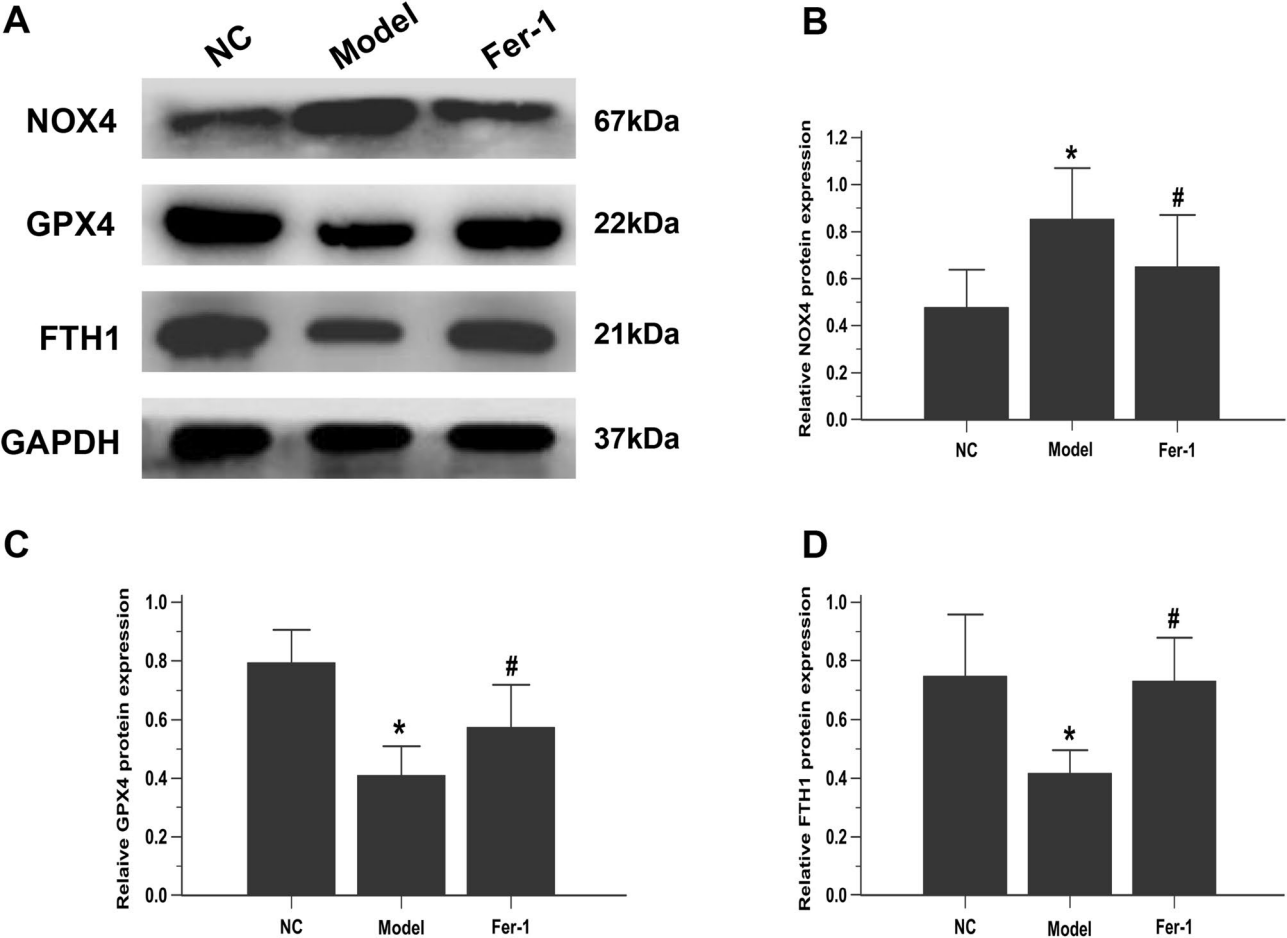
Fer-1 downregulates ferroptosis markers in PAECs. (**A**) Representative images showing the effect of Fer-1 on the expression of ferroptosis-related proteins (NOX4, GPX4 and FTH1) in PAECs, as assessed by western blot analysis. The group of blots were cropped from the same gel. (**B**–**D**) Bar graph showing that Fer-1 treatment significantly restored ferroptosis-related proteins (NOX4, GPX4 and FTH1) in PAECs. Quantitative western blot results were normalized to GAPDH. NC, normal control group; Model, model group; Fer-1, MCT + Fer-1 (10 nM) group. Each experiment was repeated 3 times, *n* = 3. The data represent the means ± SD. *P < 0.05 versus the NC group, #*P* < 0.05 versus the model group.

It is well acknowledged that GPX4, NOX4 and FTH1 are essential regulators and the most important ferroptosis markers. GPX4 is a phospholipid hydroperoxidase that protects cells against membrane lipid peroxidation^44^. NOX4, a ROS-generating enzyme, increased ferroptosis-dependent cytotoxicity by activating oxidative stress-induced lipid peroxidation^45^. Moreover, FTH1 is an iron metabolism gene involved in ferroptosis regulation. Western blotting showed that, compared to that in the NC group, NOX4 protein expression was obviously enhanced, while GPX4 and FTH1 were suppressed in the model PAEC group. As expected, these alterations were robustly inhibited by Fer-1 treatment (Fig. 2A–D). Collectively, these findings suggest that ferroptosis is present in the PAECs of MCT-induced rats.

### Fer-1 downregulates ferroptosis markers in MCT-induced PH rats

**Figure 3:**
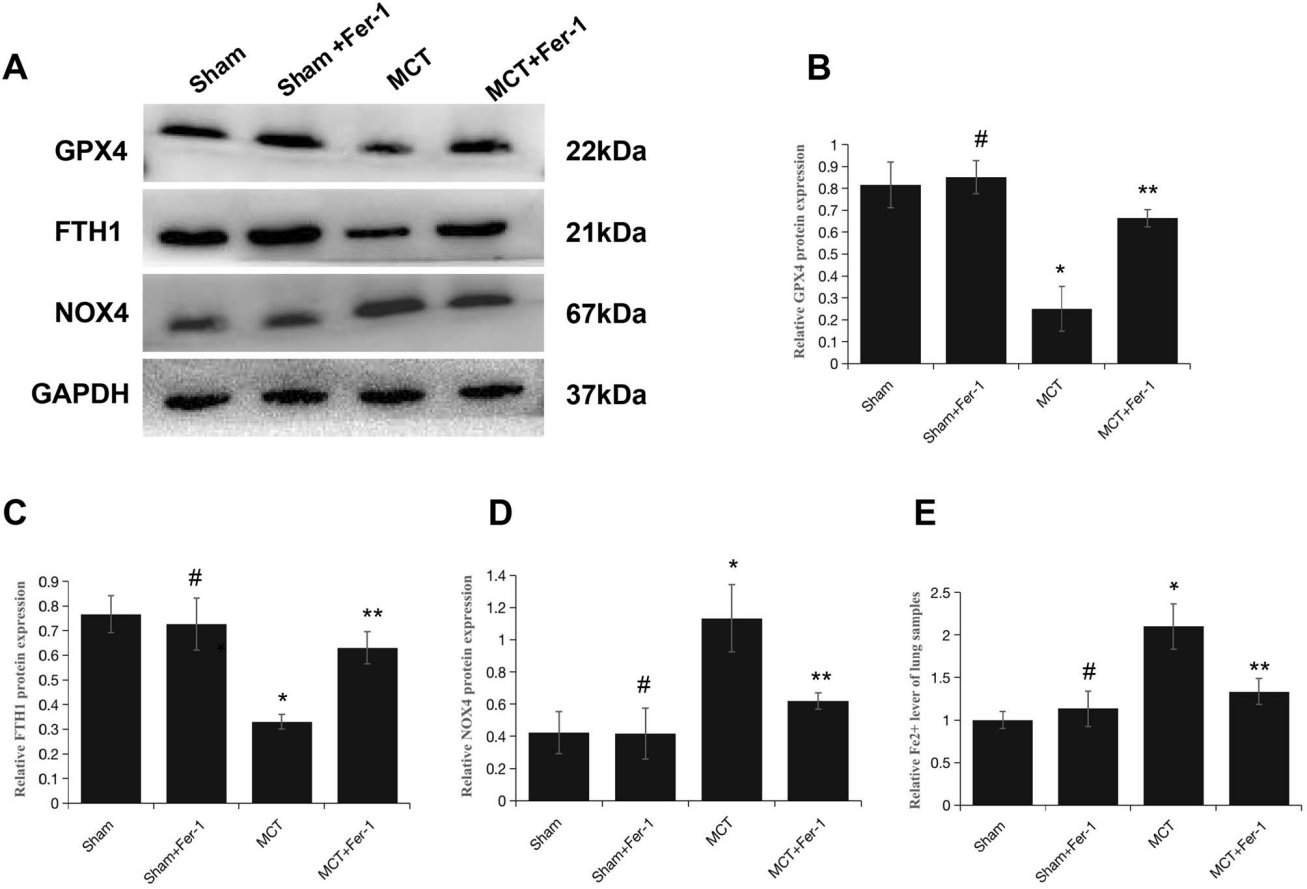
Fer-1 downregulates ferroptosis markers in MCT-induced PH rats. (**A**) Representative western blots showing ferroptosis-related proteins (NOX4, GPX4 and FTH1) in total lung homogenates from PH rats 4 weeks after MCT exposure. (**B**–**D**) The quantification of protein expression is shown; GAPDH was used as a loading control (37 kDa). The groups of blots were cropped from the same gel. (**E**) Bar graph showing Fer-1-mediated downregulation of the level of Fe^2+^ in MCT-induced PH rats. The data are shown as the mean ± SD, #*P* > 0.05 versus the sham group, *P < 0.05 versus the sham group, **P < 0.05 versus the MCT group. n = 8–12/group.

To evaluate the involvement of ferroptosis in MCT-induced PH, Fer-1 was administered to the rats. On week 4 after MCT injection, 4 rats died in the MCT group, 1 rat died in the MCT + Fer-1 group, and the rats in the sham group and sham + Fer-1 group survived to the end of the study. The relative levels of ferroptosis markers (GPX4, FTH1 and NOX4) and Fe^2+^ in lung samples were assessed. Western blot analysis demonstrated that the protein expression levels of GPX4 and FTH1 were decreased, and NOX4 expression was increased in MCT-induced rats compared with sham group rats. Moreover, the iron assay showed an increase in Fe^2+^ levels in MCT-induced rats. Fer-1 treatment inhibited these alterations in MCT-induced rats but not in the sham + Fer-1 group (Fig. 3A–E).

### Effects of Fer-1 on haemodynamics, the pulmonary vasculature and RV remodelling

**Figure 4:**
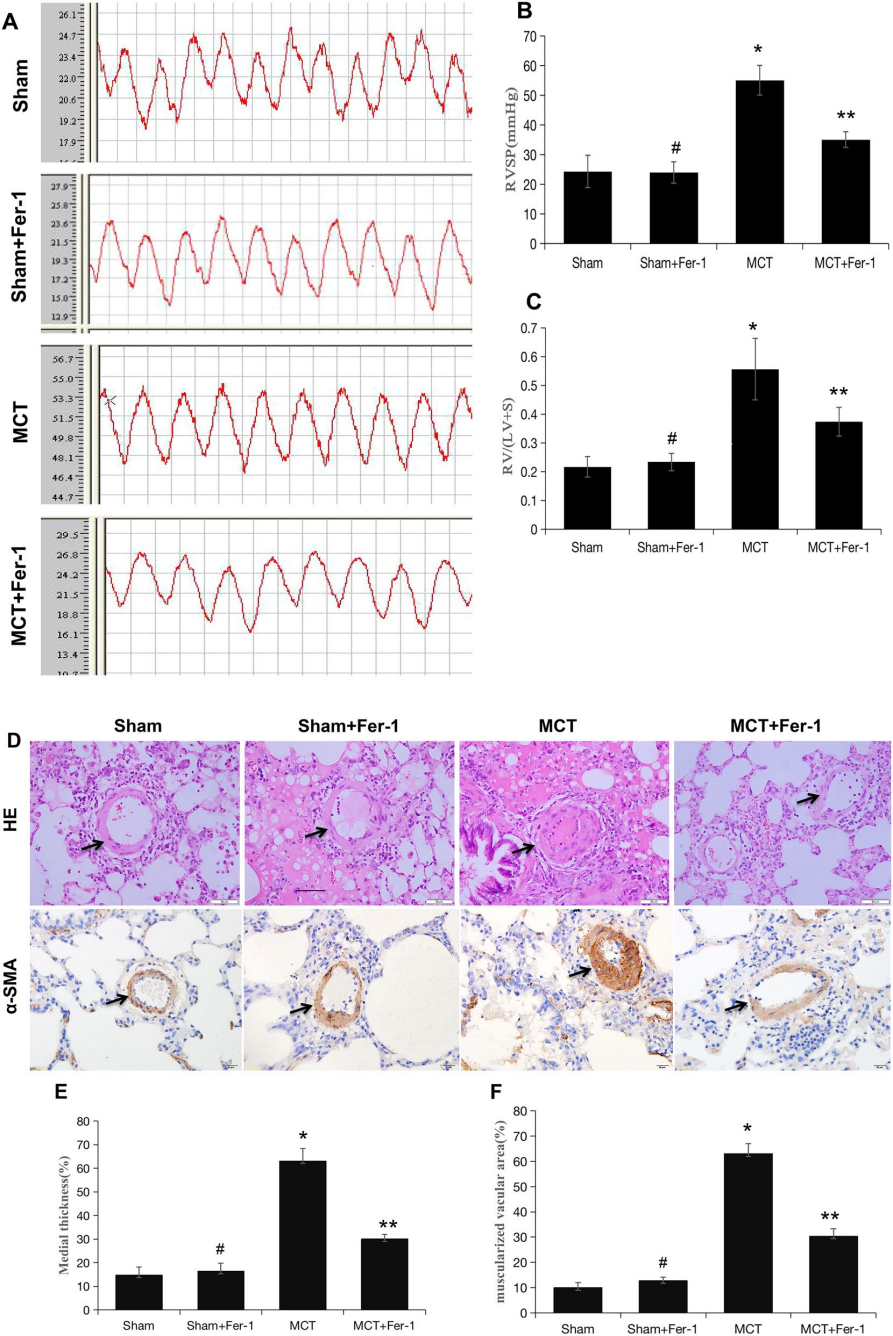
Fer-1 ameliorated MCT-induced haemodynamics, RV hypertrophy and pulmonary vascular remodelling in rats. (**A**) Representative RV pressure curves in the sham, sham + Fer-1, MCT, and MCT + Fer-1 groups. The MCT + Fer-1 group was pretreated with Fer-1 (2 mg/kg/d). (**B**) Bar graph comparing RVSPs in the different groups of rats. (**C**) Fer-1 pretreatment reduced the RV/LV + S ratio of MCT-induced rats. (**D**) Representative image of haematoxylin–eosin (HE) staining showing the expression of α-SMA to compare arteriole wall thickness in the sham, sham + Fer-1, MCT, and MCT + Fer-1 pretreatment groups. (**E**) Quantification of arteriole percent medial thickness. (**F**) The percentage of muscularization is shown by staining for α-SMA. The data represent the means ± SD. #*P* > 0.05 versus the sham group, *P < 0.05 versus the sham group, **P < 0.05 versus the MCT group. n = 8–12/group.

Consistent with previous findings^49^, MCT-induced rats developed severe PH, as reflected by a significant increase in RVSP in comparison to that in the sham group, and Fer-1 treatment effectively inhibited the MCT-induced increase in RVSP (*P* < 0.05) (Fig. 4A and B). In addition, compared with those in the MCT group, the percentage of medial wall thickness and muscularization of peripheral pulmonary arteries were markedly reduced in the MCT + Fer-1 group (*P* < 0.01), and no alterations were observed in the sham + Fer-1 group (Fig. 4D–F).

**Figure 5:**
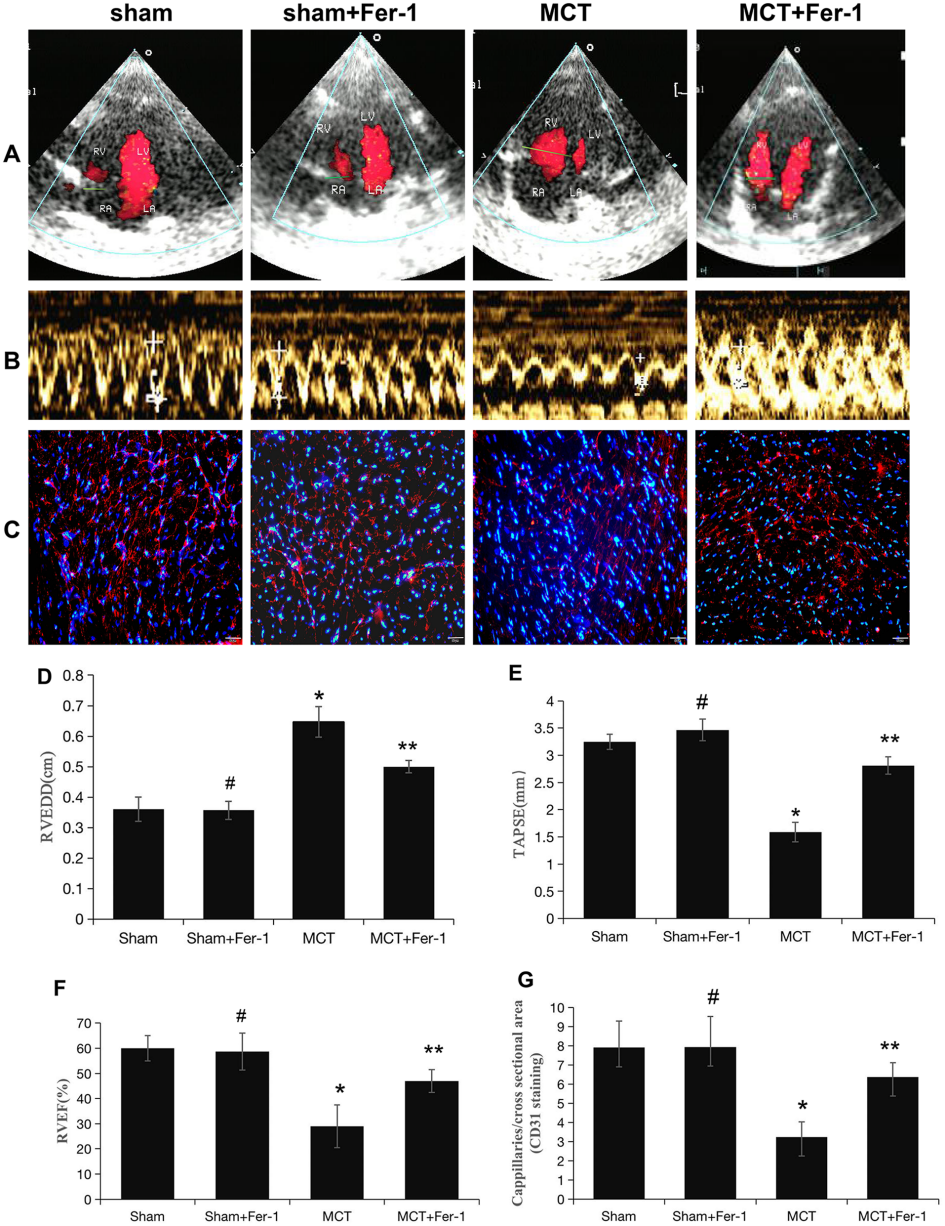
Pretreatment with Fer-1 attenuates RV function and capillaries of the RV in MCT-induced rats. (**A**, **B**) Representative colour Doppler images of the four chambers and TAPSE in the right ventricle in the sham, sham + Fer-1, MCT, and MCT + Fer-1 groups. (**C**) Representative microphotographs of right ventricles that were stained with DAPI (blue) and immunostained for CD31 (red). (**D**-**F**) Quantification of RVEDD, TAPSE, and RVEF. (**G**) Bar graph showing the numbers of capillaries in the right ventricles of the different groups of rats. The values represent the mean ± SD. #*P* > 0.05 versus the sham group, *P < 0.05 versus the sham group, **P < 0.05 versus the MCT group. *n* = 8–12/group.

Increased RV/(LV + S) ratios were also found in MCT-induced rats (Fig. 4C), indicating RV hypertrophy. Moreover, echocardiography demonstrated a significant increase in the RVEDD and decreases in TAPSE and RVEF in MCT-induced rats compared with rats in the sham group; Fer-1 treatment retarded these changes in the MCT group (*P* < 0.05), while no effects were observed in the sham + Fer-1 group (*P* > 0.05) (Fig. 5A, B, D-F). Additionally, the decrease in the number of capillaries marked by CD31 in MCT-induced rats was prevented by Fer-1 treatment (*P* < 0.05), and no changes were observed in the sham + Fer-1 group (*P* > 0.05) (Fig. 5C and G). In conclusion, ferroptosis is present in MCT-induced PH rats and is involved in the progression of PH.

### PAEC ferroptosis induces HMGB1 release, upregulates macrophage TLR4 expression and activates the NLRP3 inflammasome in vitro

**Figure 6:**
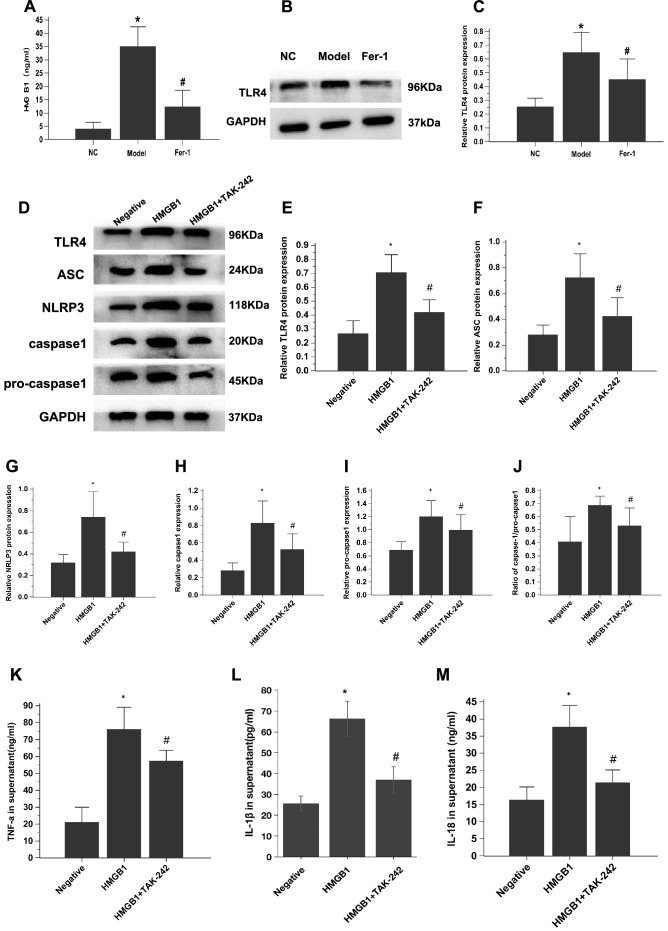
PAEC ferroptosis induces HMGB1 release, upregulates macrophage TLR4 expression and activates the NLRP3 inflammasome in vitro. (**A**) Bar graph showing that Fer-1 treatment significantly inhibited HMGB1 release in PAECs derived from MCT-induced rats. (**B**) Representative images showing the effect of Fer-1 on TLR4 expression in cocultured macrophages (NR8383 cell line). (**C**) Bar graph showing that Fer-1 treatment significantly restored TLR4 protein expression in cocultured macrophages. (**D**-**J**) TAK-242 significantly inhibited the increase in TLR4 and restored the upregulated expression of NLRP3 inflammasome-related proteins (ASC, NLRP3, pro-caspase1, and caspase1 and ratio of caspase1/pro-caspase1). The group of blots were cropped from the same gel. (**K**-**M**) Effects of TAK-242 on the secretion of TNF-α, IL-1β, and IL-18 in cocultured macrophages. Each experiment was repeated 3 times, *n* = 3. HMGB1, High-mobility group box-1 protein. *P < 0.05 versus the negative group; #*P* < 0.05 versus the HMGB1 group.

Inflammation plays an important role in the pathogenesis of PH^50^. Previous studies have shown that ferroptotic cells release HMGB1, a key DAMP that alerts the innate immune system^8, 51^. Hence, we sought to investigate the relationship between PAEC ferroptosis and HMGB1. The ELISA results showed that HMGB1 was nearly absent in the cell culture supernatant in the NC group, while HMGB1 was obviously enhanced in the model group. However, HMGB1 levels were suppressed when ferroptosis was inhibited with Fer-1 (*P* < 0.05) (Fig. 6A).

Extracellular HMGB1 induces inflammatory responses by directly acting on pattern recognition receptors, including TLR4, in different cell types, such as macrophages and neutrophils^52, 53^. Macrophages are a key driver of pulmonary remodelling; thus, TLR4 in macrophages was examined when PAECs and macrophages were cocultured. TLR4 was obviously enhanced in the model group of cocultured macrophages. Fer-1 treatment decreased the promotion of TLR4 in the model group (*P* < 0.05) (Fig. 6B and C).

The NLRP3 inflammasome plays a crucial role in the innate immune system. Recent studies have shown that the NLRP3 inflammasome is involved in PH. Inhibiting HMGB1 could improve intestinal inflammation in necrotizing enterocolitis by inhibiting NLRP3 via the TLR4 signalling pathway^53^. Thus, the present study further investigated whether TLR4 was involved in NLRP3 inflammasome activation in macrophages. HMGB1 treatment increased macrophage expression of TLR4 and NLRP3 inflammasome markers (NLRP3, ASC, pro-caspase1, caspase1 and the ratio of caspase1/pro-caspase1) and the release of inflammatory cytokines (IL-1β, IL-18 and TNF-α), while treatment with the TLR4 antagonist TAK-242 induced the opposite effects (Fig. 6D–M). These data indicate that ferroptosis regulates the progression of PH through the HMGB1/TLR4/NLRP3 inflammasome signalling pathway in vitro.

### Fer-1 downregulates HMGB1 and TLR4 expression and suppresses the NLRP3 inflammasome and proinflammatory cytokines in MCT-induced rats

**Figure 7:**
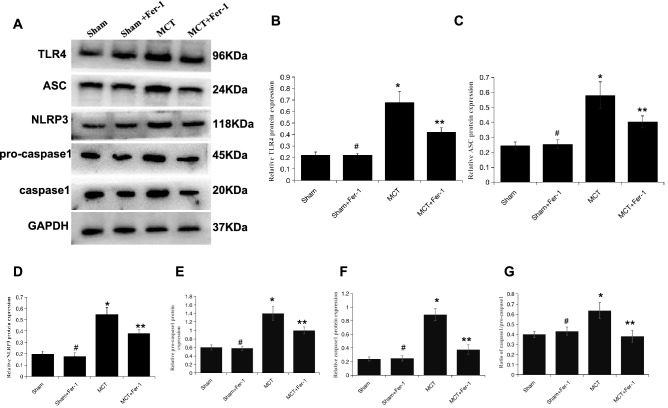
Fer-1 pretreatment inhibits lung TLR4/NLRP3 inflammasome signalling activation in MCT-induced rats. (**A**) Representative western blot showing the expression of TLR4 and NLRP3 inflammasome markers (ASC, NLRP3, pro-caspase1, caspase1) in lung samples from the different groups of rats. The groups of blots were cropped from the same gel. (**B**-**G**) Bar graph showing Fer-1-mediated downregulation of TLR4 and NLRP3 inflammasome markers (ASC, NLRP3, pro-caspase1, caspase1 and ratio of caspase1/pro-caspase1) expression in MCT-induced PH rats. Relative protein levels were determined after normalization to GAPDH. The data represent the means ± SD. #*P* > 0.05 versus the sham group, *P < 0.05 versus the sham group, **P < 0.05 versus the MCT group. *n* = 8–12/group.

**Figure 8:**
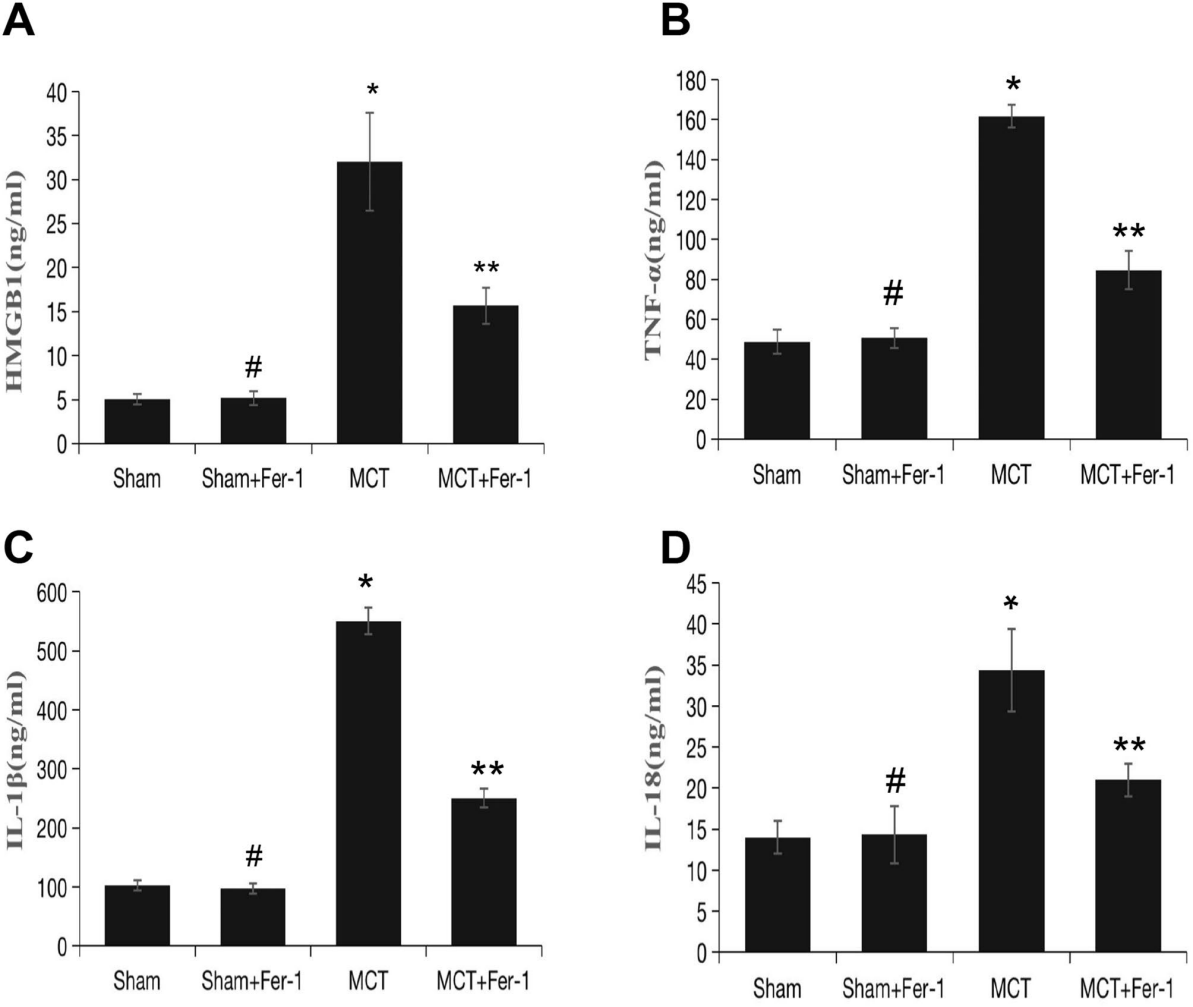
Effect of Fer-1 on HMGB1 expression and inflammatory status in rats. (**A**) Bar graph showing Fer-1-mediated inhibition of HMGB1 expression in the lung. (**B**-**D**) Tumour necrosis factor-α (TNF-α), interleukin-1β (IL-1β) and interleukin-18 (IL-18) in lung tissue. The data represent the means ± SD. #*P* > 0.05 versus the sham group, *P < 0.05 versus the sham group, **P < 0.05 versus the MCT group. *n* = 8–12/group.

Subsequently, we conducted in vivo experiments of the role of ferroptosis in the regulation of HMGB1/TLR4/NLRP3 inflammasome signalling. The ELISA results showed that, compared to that in the sham group, HMGB1 was obviously enhanced in MCT-induced rats and was suppressed by Fer-1 treatment (*P* < 0.05) (Fig. 8A). Compared with that in sham rats, TLR4 and NLRP3 inflammasome marker (NLRP3, ASC, pro-caspase1, caspase1 and ratio of caspase1/pro-caspase1) levels increased significantly in MCT rats. Fer-1 treatment of MCT-induced PH rats decreased TLR4 and NLRP3 inflammasome markers (Fig. 7A–G). Moreover, proinflammatory cytokine (including IL-1β, IL-18 and TNF-α) levels in lung homogenates from rats that were treated with Fer-1 were significantly reduced compared with those in the sham group (Fig. 8B–D).

## Discussion

To our knowledge, this is the first study to demonstrate the role of PAEC ferroptosis in MCT-induced PH and PH pathogenesis. Our results indicated the following: (1) PAEC ferroptosis, which is characterized by increased lipid peroxidation, cellular iron levels, mitochondrial damage, and abnormal ferroptosis-related proteins (GXP4, FTH1 and NOX4), plays a critical role in the progression of PH. (2) Pharmacological inhibition of ferroptosis with Fer-1 attenuated the progression of MCT-induced lung vascular remodelling and protected the right ventricle against PH. (3) PAEC ferroptosis mediates pulmonary artery remodelling via the release of HMGB1 and the subsequent engagement of TLR4, thus activating the NLRP3 inflammasome and stimulating inflammatory factor expression, which are involved in the progression of MCT-induced PH.

An expanding body of knowledge has revealed that inflammatory processes are inextricably linked to altered vascular and inflammatory cell metabolism^54^. Thus, there is a strong need to identify factors that predispose patients to impaired resolution of inflammation and to determine how immune-mediated vascular injury initiates. Based on clinical and animal studies, there is new evidence to suggest that advanced vascular remodelling may be reversed by approaches that address specific inflammatory and immune processes^50^.

Ferroptosis is an iron-dependent, nonapoptotic form of cell death that is characterized by intracellular ROS accumulation, and it is morphologically, biochemically, and genetically distinct from pyroptosis and apoptosis^5, 55^. Ferroptosis is classified as regulated necrosis and is more immunogenic than apoptosis. Previous studies have shown that PAEC apoptosis^40^ and pulmonary arterial smooth muscle cell pyroptosis are involved in pulmonary vascular inflammation^56^. Compelling evidence indicates that ferroptosis plays an important role in inflammation, and several antioxidants that act as ferroptosis inhibitors have been shown to exert anti-inflammatory effects in experimental models of different diseases^7^. Moreover, endothelial ferroptosis has been reported to be involved in atherosclerosis^57^. Consistent with previous findings, our study showed that PAEC ferroptosis presented in MCT-induced PH. Inhibiting ferroptosis with Fer-1 inhibits cell death in models of ischaemia/reperfusion injury, secondary brain injury, kidney dysfunction and radiation-induced lung fibrosis. In accordance with these findings, the present study showed that Fer-1 inhibited PAEC ferroptosis and limited the development and progression of PH in MCT-induced PH rats.

Pyroptosis, another inflammatory form of programmed cell death is involved in the immune response and has been clearly demonstrated. A previous study showed that glioma-associated oncogene family zinc finger 1 may promote pulmonary artery smooth muscle cell (PASMC) pyroptosis through ASC expression by binding with its promoter to affect the progression of PH^56^. This finding showed that pyroptosis occurred in the media of pulmonary arteries, while our study showed that ferroptosis occurred in the inner layer. An increase in ROS (either external or generated by the cell) is one initiator of PAEC dysfunction involved in PH^58, 59^. ROS may also lead to alterations in cellular proteins (such as GPX4, FTH1 and NOX4) and are a major cause of ferroptosis. Therefore, ferroptosis and pyroptosis in PAECs and PASMCs, respectively, may activate the NLRP3 inflammasome by different cellular processes associated with pulmonary vascular pathological changes, which leads to vascular remodelling in PH.

The exact regulatory mechanisms of ferroptosis in PH have not yet been fully elucidated. A previous study demonstrated that ischaemia/reperfusion injury (IRI)-induced immune cell infiltration of the cremaster muscle was mediated by ferroptosis, and Fer-1 directly inhibited leukocyte transmigration or created a milder proinflammatory microenvironment, which may have been caused by a decrease in DAMP release secondary to the decrease in ferroptosis^11, 60^. HMGB1 is a critical DAMP that is released by ferroptotic cells^8, 18^. Consistent with these findings, our current in vitro data indicated that HMGB1 was released from PAECs and that Fer-1 inhibited PAEC ferroptosis, thus decreasing HMGB1 levels. An increase in HMGB1 has also been observed in previous studies of humans and other PH models^23, 61, 62^. Similar findings in other animal models, such as doxorubicin-induced cardiomyopathy^63^ and UVB-induced skin inflammation^64^, showed that ferroptosis is involved in the progression of diseases by regulating HMGB1. The effects of HMGB1 can be mediated via several mechanisms, including HMGB1 binding to the receptor for advanced glycation end products and TLR4^53^. In line with previous findings, TLR4 expression was significantly increased in macrophages cocultured with PAECs from the MCT group compared with PAECs from the NC group.

In macrophages, activated NLRP3 couples with ASC and caspase1 to form a multiprotein cytosolic complex known as the NLRP3 inflammasome. The NLRP3 inflammasome plays a crucial role in the innate immune system. An activated NLRP3 inflammasome leads to the activation of caspase1, which subsequently promotes the maturation of IL-1β and IL-18. IL-1β and IL-18 further trigger inflammatory responses through a complex array of downstream signalling pathways, leading to a vicious cycle of inflammation^24^. Inhibiting NLRP3 signalling to decrease lung inflammation has been reported to alleviate the progression of PH in previous studies^24, 25, 65^. TLR4 provides the initial signal and leads to the upregulation of the NLRP3 inflammasome, which has been shown to be involved in different inflammation-related diseases, such as myocardial IRI^66^ and necrotizing enterocolitis^53^. In this study, the protein expression of NLRP3 was inhibited by TLR4 inhibition in macrophages, while Fer-1 inhibited the ferroptosis-induced upregulation of TLR4 and the NLRP3 inflammasome, indicating that upregulation of the TLR4/NLRP3 inflammasome is involved in MCT-induced PH.

Some limitations in our study should be mentioned. First, our results should be further investigated in other PH models, such as hypoxia or hypoxia/SU5416 and high-flow PH. Second, our observations were obtained in a rat model. Differences in pathophysiology between rats and humans require that our findings be confirmed in clinical studies.

## Conclusions

Our findings indicate that pulmonary artery endothelial ferroptosis triggers the NLRP3 inflammasome and the initial inflammatory response via the HMGB1/TLR4 pathway in MCT-induced rats. Based on these findings, treating PH with a ferroptosis inhibitor (such as Fer-1) or exploring new medicines based on ferroptosis regulation might inactivate the NLRP3 inflammasome and prevent the release of inflammatory factors, thus attenuating the progression of PH. This strategy might be promising for treating PH in the future.

## Ethics approval and informed consent

All studies were conducted according to the Guidelines for the Care and Use of Experimental Animals (NIH Publication No. 85–23, revised 1996), approved by the Guangxi Medical University Animal Ethics Committee, and carried out in compliance with the ARRIVE guidelines (http://www.nc3rs.org.uk/page.asp?id=1357).

## Consent for publication

Not applicable.

## Supplementary Information

Supplementary Information.

## Data Availability

The datasets supporting the conclusions of this article are included within the article and its supporting file.

## Abbreviations

PH: Pulmonary hypertension
MCT: Monocrotaline
PAECs: Pulmonary artery endothelial cells
GPX4: Glutathione peroxidase 4
FTH1: Ferritin heavy chain 1
LIP: Labile iron pool
NOX4: NADPH oxidase-4
Fer-1: Ferrostatin-1
HMGB1: High-mobility group box 1
TLR4: Toll-like receptor 4
NLRP3: NOD-like receptor family pyrin domain containing 3
ROS: Reactive oxygen species
DAMPs: Danger-associated molecular patterns
IL-1β: Interleukin-1β
ELISA: Enzyme-linked immunosorbent assay
TEM: Transmission electron microscopy
PASP: Pulmonary artery systolic pressure
LV: Left ventricle
RV: Right ventricular
FBS: Foetal bovine serum

## References

[1] Hoeper MM (2017). Pulmonary Hypertension. Dtsch Arztebl Int.

[2] Guignabert C (2015). New molecular targets of pulmonary vascular remodeling in pulmonary arterial hypertension: importance of endothelial communication. Chest.

[3] Thenappan T, Ormiston ML, Ryan JJ, Archer SL (2018). Pulmonary arterial hypertension: Pathogenesis and clinical management. BMJ.

[4] Lajoie AC (2016). Combination therapy versus monotherapy for pulmonary arterial hypertension: A meta-analysis. Lancet Respir. Med..

[5] Stockwell BR (2017). Ferroptosis: A regulated cell death nexus linking metabolism, redox biology, and disease. Cell.

[6] Xie Y (2016). Ferroptosis: process and function. Cell Death Differ..

[7] Sun Y (2020). The emerging role of ferroptosis in inflammation. Biomed. Pharmacother..

[8] Wen Q, Liu J, Kang R, Zhou B, Tang D (2019). The release and activity of HMGB1 in ferroptosis. Biochem. Biophys. Res. Commun..

[9] Sarhan M, von Massenhausen A, Hugo C, Oberbauer R, Linkermann A (2018). Immunological consequences of kidney cell death. Cell Death Dis..

[10] Tonnus W (2019). The clinical relevance of necroinflammation-highlighting the importance of acute kidney injury and the adrenal glands. Cell Death Differ..

[11] Li W (2019). Ferroptotic cell death and TLR4/Trif signaling initiate neutrophil recruitment after heart transplantation. J. Clin. Invest..

[12] Siques P, Brito J, Pena E (2018). Reactive oxygen species and pulmonary vasculature during hypobaric hypoxia. Front Physiol..

[13] Knock GA (2019). NADPH oxidase in the vasculature: Expression, regulation and signalling pathways; role in normal cardiovascular physiology and its dysregulation in hypertension. Free Radic. Biol. Med..

[14] Incalza MA (2018). Oxidative stress and reactive oxygen species in endothelial dysfunction associated with cardiovascular and metabolic diseases. Vascul. Pharmacol..

[15] Li L (2021). Effect of endothelial progenitor cell-derived extracellular vesicles on endothelial cell ferroptosis and atherosclerotic vascular endothelial injury. Cell Death Discov..

[16] Zhang Z (2020). The protective effects of MSC-EXO against pulmonary hypertension through regulating Wnt5a/BMP signalling pathway. J. Cell Mol. Med..

[17] Yu Y (2015). The ferroptosis inducer erastin enhances sensitivity of acute myeloid leukemia cells to chemotherapeutic agents. Mol. Cell Oncol..

[18] Zhu M (2021). Immunogenic Cell Death Induction by Ionizing Radiation. Front Immunol..

[19] Pellegrini L (2019). HMGB1 and repair: focus on the heart. Pharmacol. Ther..

[20] Paudel YN (2018). HMGB1: A common biomarker and potential target for TBI, neuroinflammation, epilepsy, and cognitive dysfunction. Front Neurosci..

[21] Aldabbous L (2016). Neutrophil extracellular traps promote angiogenesis: Evidence from vascular pathology in pulmonary hypertension. Arterioscler. Thromb. Vasc. Biol..

[22] Jia D (2017). RAGE-mediated extracellular matrix proteins accumulation exacerbates HySu-induced pulmonary hypertension. Cardiovasc. Res..

[23] Dai M (2019). HMGB1 is mechanistically essential in the development of experimental pulmonary hypertension. Am. J. Physiol. Cell Physiol..

[24] Pasqua T, Pagliaro P, Rocca C, Angelone T, Penna C (2018). Role of NLRP-3 inflammasome in hypertension: A potential therapeutic target. Curr. Pharm. Biotechnol..

[25] Lee S, Suh GY, Ryter SW, Choi AM (2016). Regulation and function of the nucleotide binding domain leucine-rich repeat-containing receptor, pyrin domain-containing-3 inflammasome in lung disease. Am. J. Respir. Cell Mol. Biol..

[26] Cero FT (2015). Absence of the inflammasome adaptor ASC reduces hypoxia-induced pulmonary hypertension in mice. Am. J. Physiol. Lung Cell Mol. Physiol..

[27] Deng Y (2019). Activation of Nicotinic Acetylcholine α7 receptor attenuates progression of monocrotaline-induced pulmonary hypertension in rats by downregulating the NLRP3 inflammasome. Front. Pharmacol..

[28] Hill NS, Gillespie MN, McMurtry IF (2017). Fifty years of monocrotaline-induced pulmonary hypertension: What Has It Meant to the Field?. Chest.

[29] Gomez-Arroyo JG (2012). The monocrotaline model of pulmonary hypertension in perspective. Am. J. Physiol. Lung Cell Mol. Physiol..

[30] Zimmer A (2021). The progression of pulmonary arterial hypertension induced by monocrotaline is characterized by lung nitrosative and oxidative stress, and impaired pulmonary artery reactivity. Eur. J. Pharmacol..

[31] Wang M (2021). S-Nitroso-L-Cysteine Ameliorated Pulmonary Hypertension in the MCT-Induced Rats through Anti-ROS and Anti-Inflammatory Pathways. Oxid. Med. Cell Longev..

[32] Zilka O (2017). On the Mechanism of Cytoprotection by Ferrostatin-1 and Liproxstatin-1 and the Role of Lipid Peroxidation in Ferroptotic Cell Death. ACS Cent. Sci..

[33] Miotto G (2020). Insight into the mechanism of ferroptosis inhibition by ferrostatin-1. Redox Biol.

[34] Sun Y, Zheng Y, Wang C, Liu Y (2018). Glutathione depletion induces ferroptosis, autophagy, and premature cell senescence in retinal pigment epithelial cells. Cell Death Dis..

[35] Xie BS (2019). Inhibition of ferroptosis attenuates tissue damage and improves long-term outcomes after traumatic brain injury in mice. CNS Neurosci. Ther..

[36] Zhang Z (2018). Glutathione peroxidase 4 participates in secondary brain injury through mediating ferroptosis in a rat model of intracerebral hemorrhage. Brain Res..

[37] Deng Y (2017). Altered mTOR and Beclin-1 mediated autophagic activation during right ventricular remodeling in monocrotaline-induced pulmonary hypertension. Respir. Res..

[38] Kamezaki F (2008). Gene transfer of extracellular superoxide dismutase ameliorates pulmonary hypertension in rats. Am. J. Respir. Crit. Care Med..

[39] Homma N (2008). Involvement of RhoA/Rho kinase signaling in protection against monocrotaline-induced pulmonary hypertension in pneumonectomized rats by dehydroepiandrosterone. Am. J. Physiol. Lung Cell Mol. Physiol..

[40] Yamaji-Kegan K (2014). Hypoxia-induced mitogenic factor (FIZZ1/RELMalpha) induces endothelial cell apoptosis and subsequent interleukin-4-dependent pulmonary hypertension. Am. J. Physiol. Lung Cell Mol. Physiol..

[41] Zuniga MC, White SL, Zhou W (2014). Design and utilization of macrophage and vascular smooth muscle cell co-culture systems in atherosclerotic cardiovascular disease investigation. Vasc. Med.

[42] Liu B (2018). Puerarin protects against heart failure induced by pressure overload through mitigation of ferroptosis. Biochem. Biophys. Res. Commun..

[43] Cao JY, Dixon SJ (2016). Mechanisms of ferroptosis. Cell Mol. Life Sci..

[44] Hao S (2017). Cysteine Dioxygenase 1 Mediates Erastin-induced ferroptosis in human gastric cancer cells. Neoplasia.

[45] Li Y (2020). Inhibitor of apoptosis-stimulating protein of p53 inhibits ferroptosis and alleviates intestinal ischemia/reperfusion-induced acute lung injury. Cell Death Differ..

[46] Mao X-Y, Zhou H-H, Jin W-L (2019). Ferroptosis induction in pentylenetetrazole kindling and pilocarpine-induced epileptic seizures in Mice. Front Neurosci-Switz.

[47] Anthonymuthu TS (2021). Resolving the paradox of ferroptotic cell death: Ferrostatin-1 binds to 15LOX/PEBP1 complex, suppresses generation of peroxidized ETE-PE, and protects against ferroptosis. Redox Biol..

[48] Yagoda N (2007). RAS-RAF-MEK-dependent oxidative cell death involving voltage-dependent anion channels. Nature.

[49] Deng Y (2021). Interferon regulatory factor 7 inhibits rat vascular smooth muscle cell proliferation and inflammation in monocrotaline-induced pulmonary hypertension. Life Sci..

[50] Rabinovitch M, Guignabert C, Humbert M, Nicolls MR (2014). Inflammation and immunity in the pathogenesis of pulmonary arterial hypertension. Circ. Res..

[51] Efimova I (2020). Vaccination with early ferroptotic cancer cells induces efficient antitumor immunity. J. Immunother. Cancer.

[52] Wang J (2020). HMGB1 participates in LPSinduced acute lung injury by activating the AIM2 inflammasome in macrophages and inducing polarization of M1 macrophages via TLR2, TLR4, and RAGE/NFkappaB signaling pathways. Int. J. Mol. Med..

[53] Yu R (2019). Inhibition of HMGB1 improves necrotizing enterocolitis by inhibiting NLRP3 via TLR4 and NF-kappaB signaling pathways. J. Cell Physiol..

[54] Sutendra G (2011). Pyruvate dehydrogenase inhibition by the inflammatory cytokine TNFalpha contributes to the pathogenesis of pulmonary arterial hypertension. J. Mol. Med. (Berl).

[55] Dixon SJ (2012). Ferroptosis: an iron-dependent form of nonapoptotic cell death. Cell.

[56] He S (2020). GLI1-mediated pulmonary artery smooth muscle cell pyroptosis contributes to hypoxia-induced pulmonary hypertension. Am. J. Physiol. Lung Cell Mol. Physiol..

[57] Bai T, Li MX, Liu YF, Qiao ZT, Wang ZW (2020). Inhibition of ferroptosis alleviates atherosclerosis through attenuating lipid peroxidation and endothelial dysfunction in mouse aortic endothelial cell. Free Radical Bio. Med..

[58] Acker SN (2015). Altered pulmonary artery endothelial-smooth muscle cell interactions in experimental congenital diaphragmatic hernia. Pediatr. Res..

[59] Sun X (2020). TGF-beta1 attenuates mitochondrial bioenergetics in pulmonary arterial endothelial cells via the disruption of carnitine homeostasis. Redox. Biol..

[60] Sarhan M, Land WG, Tonnus W, Hugo CP, Linkermann A (2018). Origin and consequences of necroinflammation. Physiol. Rev..

[61] Huang YY (2016). Elevated serum HMGB1 in pulmonary arterial hypertension secondary to congenital heart disease. Vascul. Pharmacol..

[62] Li WJ (2017). HMGB1 affects the development of pulmonary arterial hypertension via RAGE. Eur. Rev. Med. Pharmacol. Sci..

[63] Zhang H, Wang Z, Liu Z, Du K, Lu X (2021). Protective effects of dexazoxane on rat ferroptosis in doxorubicin-induced cardiomyopathy through regulating HMGB1. Front Cardiovasc. Med..

[64] Vats K (2021). Keratinocyte death by ferroptosis initiates skin inflammation after UVB exposure. Redox Biol..

[65] Villegas LR (2013). Superoxide dismutase mimetic, MnTE-2-PyP, attenuates chronic hypoxia-induced pulmonary hypertension, pulmonary vascular remodeling, and activation of the NALP3 inflammasome. Antioxid. Redox. Signal.

[66] Dai Y (2020). M2 macrophage-derived exosomes carry microRNA-148a to alleviate myocardial ischemia/reperfusion injury via inhibiting TXNIP and the TLR4/NF-kappaB/NLRP3 inflammasome signaling pathway. J. Mol. Cell. Cardiol..

