# Supplementary material for: Endothelial cell ferroptosis mediates monocrotaline-induced pulmonary hypertension in rats by modulating NLRP3 inflammasome activation
Source: Sci Rep. 2022 Feb 23;12:3056. doi: 10.1038/s41598-022-06848-7 (PMC8866506; doi:10.1038/s41598-022-06848-7)
Supplement: Supplementary file 1 — Supplementary Information. [file 41598_2022_6848_MOESM1_ESM.pdf]

fig 2A PAEC NOX4 67kDa

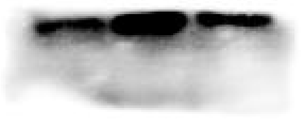

fig 2A PAEC GPX4 22kDa

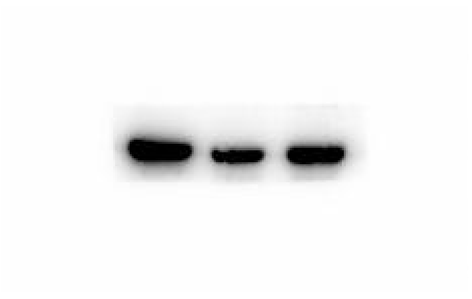

fig 2A PAEC FTH1 21kDa

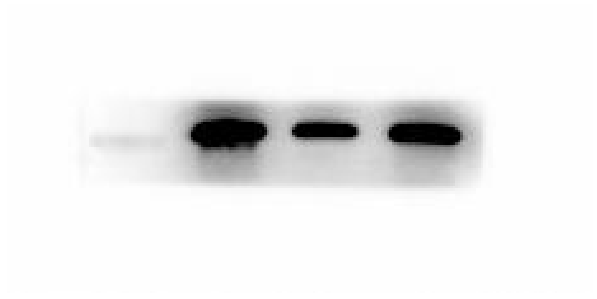

fig 2A PAEC GAPDH 37kDa

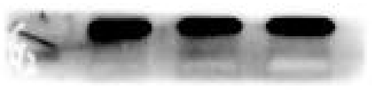

fig 3A rats NOX4 67kDa

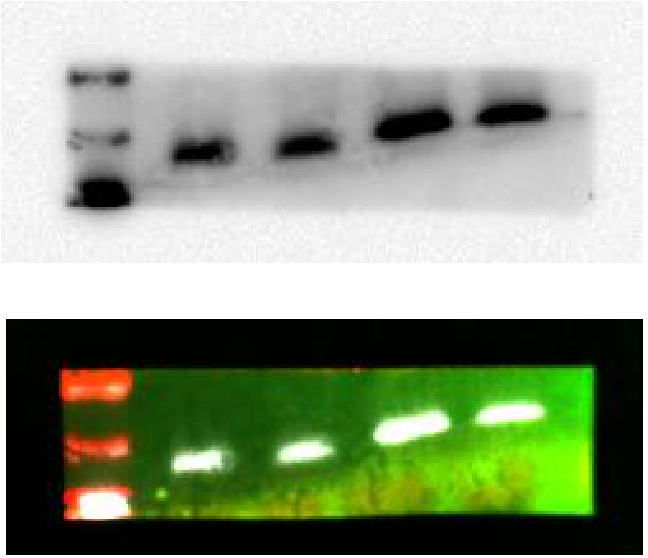

fig 3A rats GPX4 22kDa

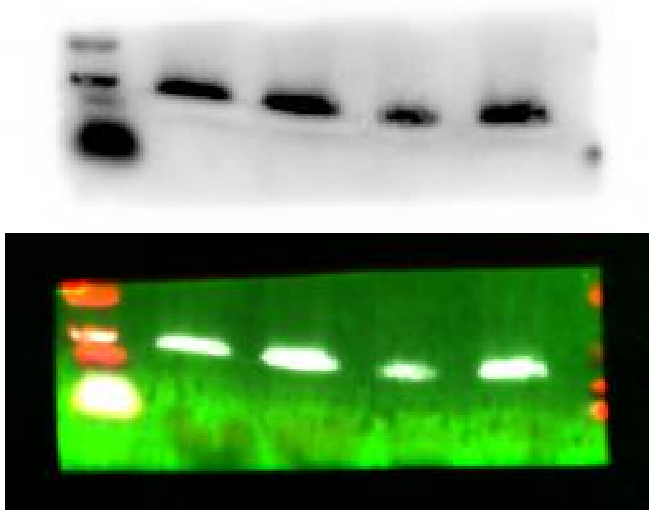

fig 3A rats FTH1 21kDa

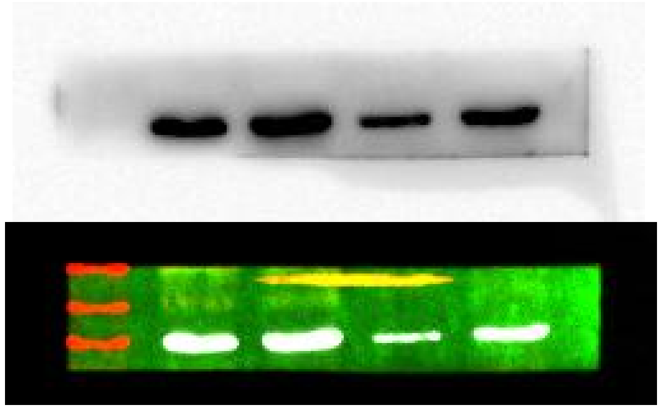

fig 3A rats GAPDH 37kDa

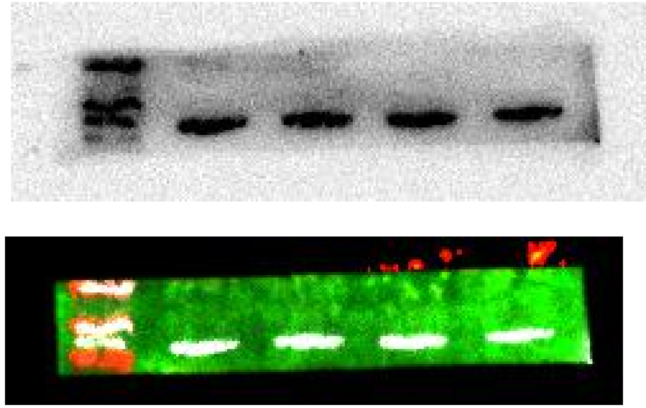

Caption:The red lines are makers which indicate the molecular weight of proteins in the gel and nitrocellulose membranes.

fig 6B macrophagy TLR4 96kDa

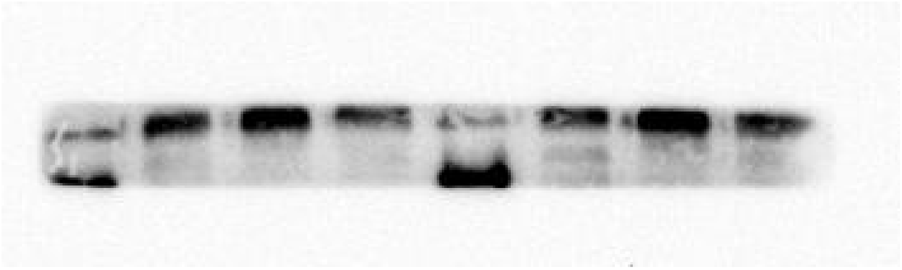

fig 6B macrophagy GAPDH 37kDa

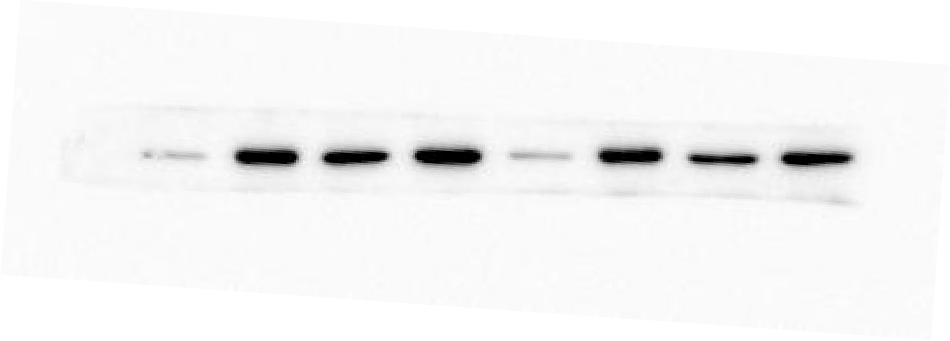

fig6D TLR4 96kDa

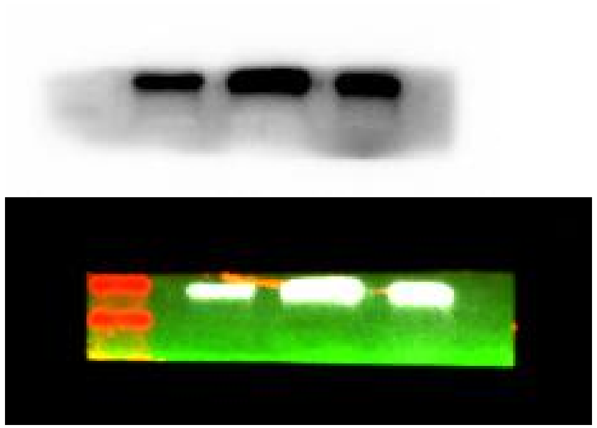

fig6D ASC 24kDa

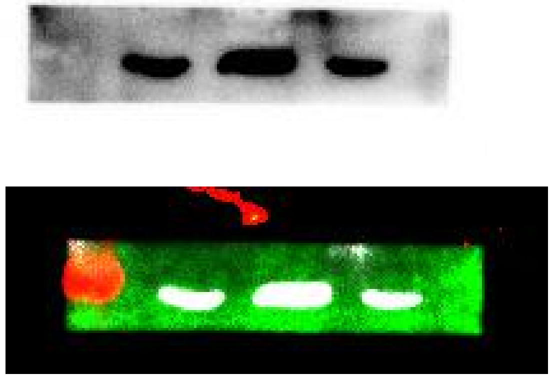

fig6D NLRP3 118kDa

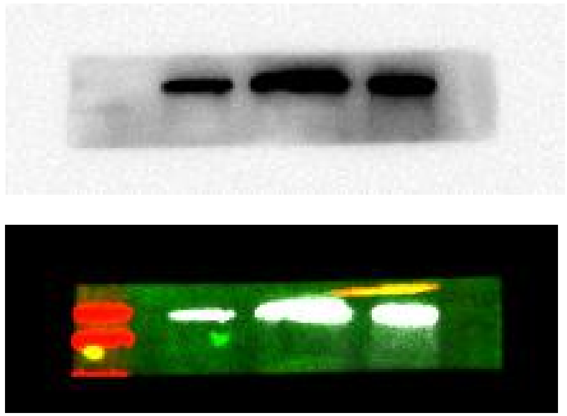

fig6D caspase1 20kDa

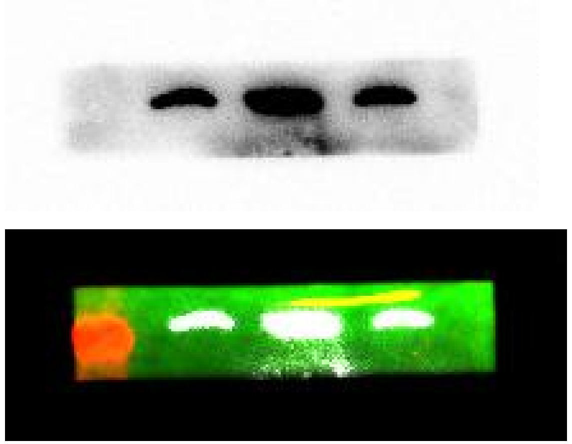

fig6D pro-caspase1 45kDa

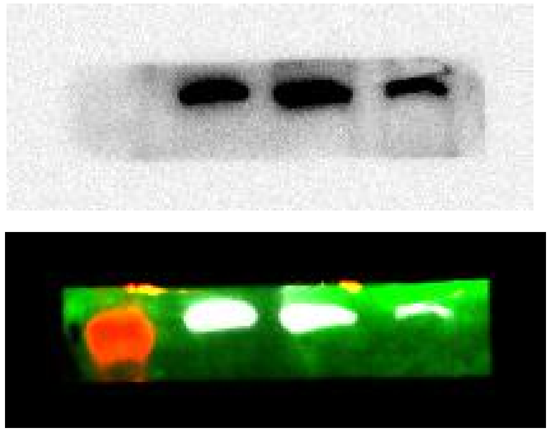

fig6D GAPDH 37kDa

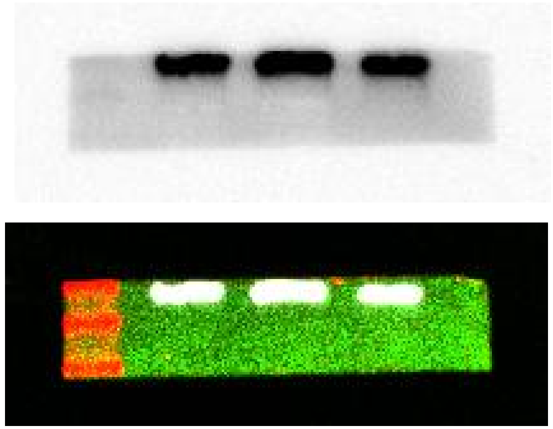

Caption:The red lines are makers which indicate the molecular weight of proteins in the gel and nitrocellulose membranes.

fig7A TLR4 96kDa

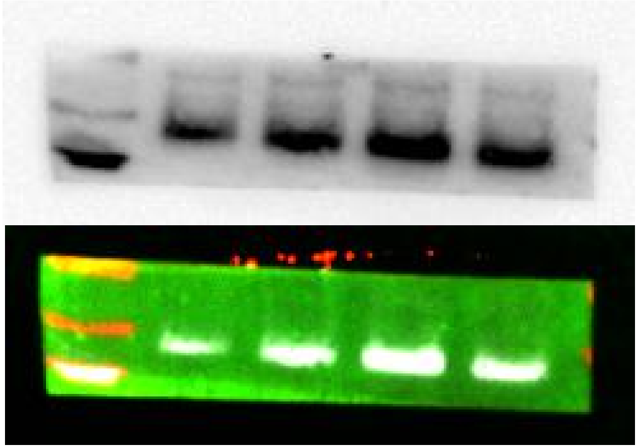

fig7A ASC 24kDa

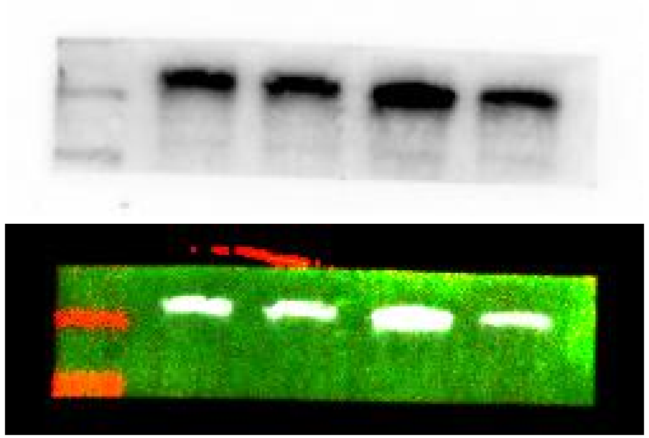

fig7A NLRP3 118kDa

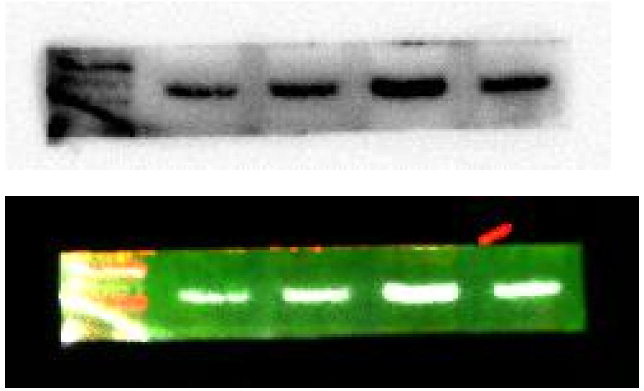

fig7A caspase1 20kDa

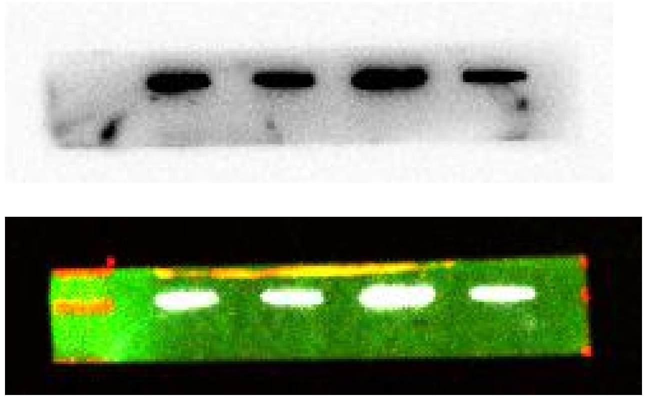

fig7A pro-caspase1 45kDa

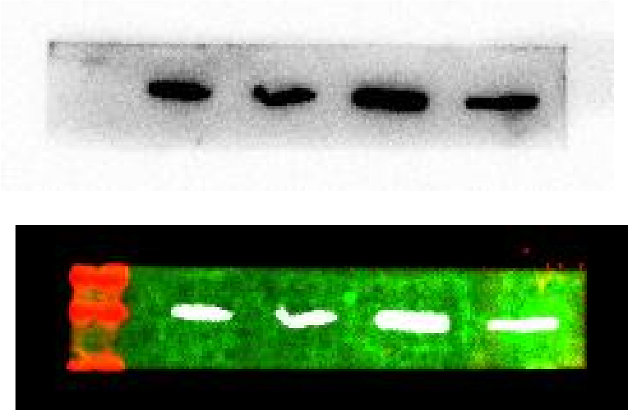

fig7A GAPDH 37kDa

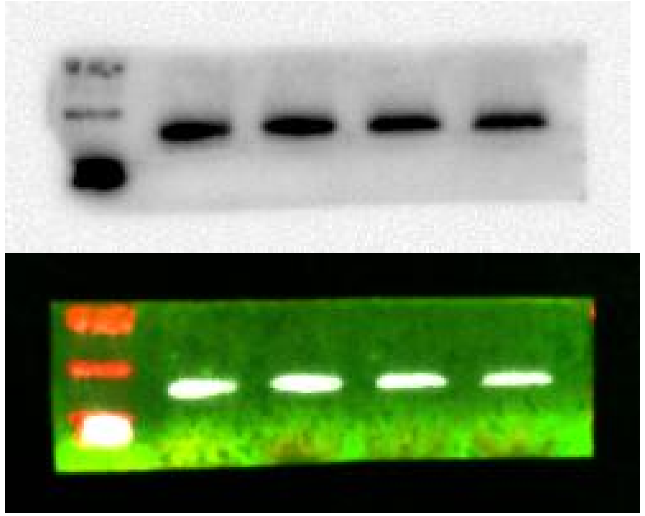

Caption:The red lines are makers which indicate the molecular weight of proteins in the gel and nitrocellulose membranes.
